# Screening rate constants in the simulation of rapid kinetics of chlorophyll *a* fluorescence using the Morris method

**DOI:** 10.3389/fpls.2024.1396309

**Published:** 2024-06-13

**Authors:** Hui Lyu, Ying-Chao Lin, Georgios Liakopoulos

**Affiliations:** ^1^ School of Biological Science and Agriculture, Qiannan Normal University for Nationalities, Duyun, China; ^2^ Guizhou Academy of Tobacco Science, Guiyang, China; ^3^ Laboratory of Plant Physiology and Morphology, Department of Crop Production, Agricultural University of Athens, Athens, Greece

**Keywords:** chlorophyll *a* fluorescence, the Morris method, sensitivity analysis, rate constant, photosynthesis model

## Abstract

Chlorophyll *a* fluorescence, a sensitive and cost-effective probe, is widely used in photosynthetic research. Its rapid phase, occurring within 1 second under intense illumination, displays complex O-J-I-P transients, providing valuable insights into various aspects of photosynthesis. In addition to employing experimental approaches to measure the rapid Fluorescence Induction (FI) kinetics, mathematical modeling serves as a crucial tool for understanding the underlying mechanisms that drive FI dynamics. However, the significant uncertainty and arbitrary nature of selecting model parameters amplify concerns about the effectiveness of modeling tools in aiding photosynthesis research. Therefore, there is a need to gain a deeper understanding of how these models operate and how arbitrary parameter choices may influence their outcomes. In this study, we employed the Morris method, a global Sensitivity Analysis (SA) tool, to assess the significance of rate constants employed in an existing fluorescence model, particularly those linked to the entire electron transport chain, in shaping the rapid FI dynamics. In summary, utilizing the insights gained from the Morris SA allows for targeted refinement of the photosynthesis model, thereby improving our understanding of the complex processes inherent in photosynthetic systems.

## 1 Introduction

When a dark-adapted photosynthetic sample is exposed to high light intensity, chlorophyll (chl) *a* fluorescence is emitted mainly from the antenna of Photosystem II (PSII). Roughly 2% to 8% (Trissl et al., 1993) of the total absorbed light energy is converted into chl *a* fluorescence, while the remaining energy is utilized for photochemical reactions and dissipated as heat. Chl *a* fluorescence is not exclusively emitted from PSII, but Photosystem I (PSI) also contributes in shaping the fluorescence intensity (Trissl et al., 1993; Pfündel, 1998). Chl *a* fluorescence has long been a widely employed probe for investigating diverse aspects of photosynthesis because fluorescence measurements are non-invasive, highly sensitive, and easy to implement (Oxborough and Baker, 1997; Baker, 2008). Most importantly, the chl *a* fluorescence signal contains information from nearly every facet of photosynthesis (Valcke, 2021). Under continuous illumination, the measured Fluorescence Induction (FI) curve exhibits a rapid initial increase within a second, followed by a decrease over the course of a few minutes. This rapid phase in FI kinetics during intense light regime is generally denoted as O-J-I-P, with the O-J rise being considered as the photochemical phase and the subsequent J-I-P phase being considered as the thermal phase (Strasser et al., 1995; Strasser, 1997). Here, O stands for the origin when Q_A_, which is the primary quinone electron acceptor of PSII, is completely oxidized at the beginning of measurements. J and I denote intermediate levels, while P denotes the peak, which, under saturating light conditions, corresponds to the maximum fluorescence (F_M_).

Despite about four decades of measuring the FI curve, the interpretation of its rapid phase still remains a complex task [for comprehensive reviews, see (Stirbet and Govindjee, 2012; Murchie and Lawson, 2013; Schansker et al., 2014; Stirbet et al., 2014, 2020; Bhagooli et al., 2021; Valcke, 2021; Janeeshma et al., 2022)]. This complexity may arise from the interrelationship of various photosynthetic processes, contributing to the characteristic variations observed in FI curves. Undoubtedly, the oxidized Q_A_ is widely recognized as the quencher for chl *a* fluorescence [for comprehensive reviews, see (Govindje, 1995; Stirbet and Govindjee, 2012; Stirbet et al., 2014, 2020; Janeeshma et al., 2022)]. Also, the oxidized molecules in the PQ pool have been shown to be responsible for quenching chl *a* fluorescence (Vernotte et al., 1979). This observation has been incorporated into various theoretical investigations (Stirbet et al., 1998; Tomek et al., 2001; Zhu et al., 2005; Lazár, 2009). Moreover, the redox states of the Oxygen-Evolving Complex (OEC) (Delosme and Joliot, 2002; Jablonsky and Lazar, 2008; Jablonsky et al., 2008), in conjunction with the activity of Cytb_6_/f (Johnson and Berry, 2021) and PSI (Schreiber and Krieger, 1999; Schansker et al., 2003; Schansker and Strasser, 2005; Schansker et al., 2005), have been shown to influence the thermal phase (J-I-P rise) of the rapid FI curve.

Additionally, Ferredoxin-NADP^+^-oxidoreductase (FNR), usually inactive in plants during dark-adaption, can become active during the rapid phase of FI curve, thereby influencing the electron transport around PSI and hence the thermal phase in specific plants (Ilík et al., 2006). Furthermore, conformational changes that impact fluorescence yield have also been proposed to occur during the rapid phase of FI curves (Magyar et al., 2018; Sipka et al., 2019, 2021; Magyar et al., 2022; Sipka et al., 2022).

In addition to experimental studies aimed at exploring the origin of FI curves, the application of mathematical models has proven to be a valuable approach for suggesting the mechanisms underlying the characteristic changes in FI curves. To simulate rapid FI curves, Renger and Schulze (1985) (Renger and Schulze, 1985) initially employed a “structure-based” Two-Electron Gate (TEG) model to simulate and fit various fluorescence transients measured under different low light intensities. The term “structure-based” indicates that the model was constructed using widely accepted structural and/or functional information of the photosynthetic system. Following that, many extended TEG models have been proposed [e.g (Stirbet et al., 1998; Lebedeva et al., 2002; Lazár, 2003; Zhu et al., 2005; Lazár, 2009; Belyaeva et al., 2016; 2019)], incorporating numerous biochemical reactions related to photosynthesis that can influence the rapid kinetics of FI curves. These “structure-based” FI models generally involve a system of interconnected ordinary differential equations (ODEs). By assigning initial values for parameters and incorporating rate constants obtained from published literature into the model, authors can determine the temporal evolution of variables under investigation. Certainly, models can quantitatively suggest the impact of parameters on the variables of interest by adjusting parameter values or setting them directly to zero (Lazár et al., 1997, 1998; Lazár and Pospíšil, 1999; Lazár, 2003; Lazár et al., 2005; Lazár, 2009, 2013). However, when the output of a variable influenced by one parameter (e.g., the rate constant in an FI model) shows more variation than its response to another rate constant, it is easy to identify which rate constant has a more pronounced impact. On the contrary, when the variable outputs induced by both rate constants are essentially indistinguishable, it becomes challenging to determine which rate constant exerts a greater influence on the investigated variable. In such a scenario, the ability of models to decipher the rapid FI curves can be limited.

In this study, we conducted Sensitivity Analysis (SA) on parameters, particularly those associated with the entire electron transport chain, of a previously published FI model (Lazár, 2009), employing the Morris method (Morris, 1991). The Morris SA method examines how the uncertainty of input parameters affects the output of model variables and is especially applicable to models with a large number of parameters (Morris, 1991; Campolongo et al., 2007). This global SA technique is widely applied in various research areas, including pasture management (Ben Touhami et al., 2013), water supply (King and Perera, 2013), waste treatment (Langergraber and Ketema, 2015), nuclear science (Wang et al., 2020), and space exploration (Sohier et al., 2014, 2015). Notably, we have observed examples where authors (Lazár et al., 2005; Ebenhoh et al., 2011; Zhu et al., 2013) utilized a basic local derivative-based SA method to screen the significance of FI parameters. This local SA technique assesses the sensitivity of model inputs solely at a specific point in the input space. Although this method is simple and computationally inexpensive, it is most informative only if the model is linear. In many cases, particularly for complex nonlinear models with numerous factors, the application of this local SA method may lead to incorrect conclusions (for comprehensive reviews, see (Borgonovo and Plischke, 2016; Qian and Mahdi, 2020)). In addition to the Morris method, other global SA techniques such as Sobol, FAST (Fourier Amplitude Sensitivity Test), and eFAST (extended Fourier Amplitude Sensitivity Test) fall under the variance-based category. These approaches prove effective for models with a moderate number of parameters. However, as the number of parameters increases, the computational complexity rises with the expanding dimensionality of the input space, leading to a significant increase in computational costs (Qian and Mahdi, 2020).

In this study, our primary objective is to address a fundamental challenge faced by fluorescence modelers: identifying which parameters, especially those linked to the electron transport chain, exert significant influence on fluorescence dynamics once the model outcomes are obtained, and ranking the importance of these parameters based on their sensitivity.

## 2 Theoretical description

### 2.1 Simulation of rapid kinetics of chlorophyll *a* fluorescence

In this study, the Morris method was used to analyze the photosynthesis model developed by Lazár (2009) [27]. Lazár’s model (2009) has several features as presented below:

1, the model consists of 43 variables, 34 rate constants, and a set of mutually coupled 43 nonlinear differential equations. This configuration clarifies the sequence of electron transport reactions, starting at OEC and progressing through PSII, the Plastoquinone pool (PQ pool), Cytb_6_/f complex, PSI, and terminating at FNR. Notably, the model integrates the functionality of FNR and the cyclic electron transport reactions from ${\text{F}}_{\text{D}}^{-}$ (reduced ferredoxin) back to Cytb_6_/f or PQ pool, providing a description of the entire electron transport reactions from OEC to FNR;

2, the model has the capability to simultaneously simulate the rapid phases of FI curves and 820 nm transmittance signals (I_820_) measured in pea leaves, not only under controlled conditions but also in samples treated with DBMIB (2,5-dibromo-3-methyl-6-isopropyl-p-benzoquinone, capable of interrupting the electron transport from the PQ pool to the Cytb_6_/f complex) and MV (1,1´dimethyl-4,4´-bipyridinium-dichloride, capable of accepting electrons originating from PSI). Furthermore, the model has the capability to simulate the rapid phases of FI curves and I_820_ signals under varying light intensities.

Until now, Lazár’s model (2009) can be considered as one of the classical models that comprehensively incorporates the entire electron transport reactions from OEC to FNR. Additionally, various other models, providing an overview of the entire photosynthetic system, have also been developed (Laisk et al., 2006; Nedbal et al., 2007; Zhu et al., 2013; Lyu and Lazár, 2017). Notably, a series of models developed by Rubin and coworkers (Lebedeva et al., 2002; Belyaeva et al., 2016; 2019) are characterized by their consideration of the impact of transthylakoid electric potential difference on regulating the rate of electron transport reactions. In this study, our focus is on identifying the reactions within the entire electron transfer chain that significantly impact fluorescence output. Therefore, Lazár’s model was chosen for our case study as it meets our specific requirements. For a more detailed theoretical description of Lazár’s model, readers can refer to the author’s original paper (Lazár, 2009).

### 2.2 The Morris method

The Morris method employs a One-At-a-Time (OAT) design that proves to be cost-effective when dealing with models with a large number of inputs. The Morris method enables the derivation of the Elementary Effect (EE) for a specific factor of interest through a finite difference scheme. For a given **X** = (X_1_,···,X_i_,···,X_j_), the EE_i_ of X_i_ can be determined using the following formula:


(1) $$EE_{i}=\frac{f(X_{1},\cdots ,X_{i}+\Delta ,\cdots ,X_{j})-f(X_{1},\cdots ,X_{i},\cdots ,X_{j})}{\Delta }$$

In **Equation 1**, the function f(**X**) represents the output generated by the simulation; the value of Δ is typically set as (p-2)/(p-1), and p is the number of levels.

In summary, an “elementary effect” refers to the extent of variation in output observed by incrementally perturbing individual parameters. Unlike traditional methods that alter one parameter at a time, Morris employs a continuous and incremental perturbation approach to explore the parameter space. This method allows for acquiring information about model parameter sensitivity at a relatively low computational cost without the need for prior sampling of the parameter space. Due to its simple and iterative nature, the Morris SA tool is widely employed for understanding model behavior and optimizing parameter selection.

#### 2.2.1 Trajectory-based sampling strategy

Morris (1991) (Morris, 1991) initially introduced the trajectory-based sampling strategy, which involves generating m trajectories, each consisting of k+1 points in the input space, where k represents the number of input factors. In each trajectory, k elementary effects are determined, with one EE corresponding to each input factor. This results in a total of m × (k+1) sample points. Conceptually, a trajectory can be considered as a matrix that initiates with a “base” value X* for the vector X. This X* is randomly chosen as the initial point for generating an entire trajectory. The trajectory matrix B’ can be constructed as follows:


(2) $$B^{\prime }=J_{k+1,1}X^{*}+\Delta B\;$$

In **Equation 2**, the term J_k+1,1_ denotes a matrix of dimensions (k+1) × 1, comprising exclusively of 1’s, X* is a randomly chosen “base value” for the vector X, and B is a strictly lower triangular matrix of 1’s.

B’ is considered as a potential candidate for the desired trajectory matrix. However, it has the limitation that the k elementary effects it generates are not randomly selected. To overcome this limitation, modifications have been made, leading to the creation of a properly randomized sampling matrix (Morris, 1991):


(3) $$B^{*}=\left(J_{k+1,1}X^{*}+\left(\Delta /2\right)\left[\left(2B-J_{k+1,k}\right)D^{*}+J_{k+1,k}\right]\right)P^{*}$$

In the given formula, J_k+1,k_ is a (k+1) × k matrix consisting of 1’s, D* is a k-dimensional diagonal matrix where each element is randomly assigned either +1 or -1 with equal probability, and P* is a k × k random permutation matrix.

#### 2.2.2 The Morris measures

In most cases, both the mean value (μ_i_) and standard deviation (σ_i_) can be determined through repetitive calculations for each input parameter. A high μ_i_ value indicates a significant impact on the output exerted by the input parameter, while a low μ_i_ value suggests minimal influence. Furthermore, a high σ_i_ value signifies nonlinear effects on the output and/or potential interactions with other parameters. These statistical measures are calculated using Equations 4, 5:


(4) $$\mu_{i}=\frac{\sum_{k=1}^{r}EE_{i}^{k}}{r}\;$$

(5) $$\sigma_{i}=\sqrt{\frac{\sum_{k=1}^{r}{\left(EE_{i}^{k}-\mu_{i}\right)}^{2}}{r-1}}$$

However, the μ_i_ value fails to account for situations in which elementary effects with opposite signs cancel each other, leading to a Type II error. This error represents the failure to identify a factor of considerable influence on the model. To overcome this limitation, Campolongo et al. (2007) (Campolongo et al., 2007) introduced a new index as expressed in Equation 6:


(6) $$\mu_{i}^{*}=\frac{\sum_{k=1}^{r}\left|EE_{i}^{k}\right|}{r}$$

The index ${\text{μ}}_{\text{i}}^{*}$ can provide more comprehensive information about the SA when combined with μ_i_ and σ_i_.

#### 2.2.3 An application example of the Morris SA method

Overall, the Morris SA offers a systematic approach to evaluate the influence of input parameters on model outputs. In its application, the first step involves identifying the key input variables within the model. Following this, the input ranges for each parameter are defined, encompassing plausible real-world values. Subsequently, sample sets are generated, comprising various combinations of input values across the defined ranges. These samples are then used to run the model, producing corresponding output data. The elementary effects of each parameter are then calculated, reflecting the change in output resulting from small variations in individual inputs while holding others constant. By averaging these elementary effects, parameters are ranked based on their sensitivity, elucidating which inputs exert the most significant impact on model outcomes. Interpretation of these results provides valuable insights into the dynamics of the model, guiding further refinements or informing decision-making processes. Here, we present an application example of the Morris SA method on a simple set of differential equations:


(7) $$\frac{dx_{1}}{dt}=ax_{1}+bx_{2}$$

(8) $$\frac{dx_{2}}{dt}=ax_{1}-bx_{2}$$

We will explore the impact of variations in x_1_ or x_2_ on the output of y = x_1_ + x_2_. Here, x_1_ and x_2_ can be thought as rate constants in the fluorescence model, while y represents the simulated variable fluorescence emission. We set the initial values of x_1_ and x_2_ to 1, a and b to 5, with both a and b scaled at 20%. The time interval t is set from 0 to 1 s, p is set to 4, and Δ to 2/3. Following Equation 3, we set the values of B, D^∗^, J, P^∗^, and X^∗^. Ultimately, we generate four matrices for sampling both a and b:


$$\text{Trajectory }1:\;\left[\begin{array}{cc} 7.3 & 6\\ 6 & 6\\ 6 & 7.3 \end{array}\right]$$

$$\text{Trajectory }2:\;\left[\begin{array}{cc} 6 & 5.3\\ 4.6 & 5.3\\ 4.6 & 6.6 \end{array}\right]$$

$$\text{Trajectory }3:\;\left[\begin{array}{cc} 6.6 & 4.6\\ 5.3 & 4.6\\ 5.3 & 6 \end{array}\right]$$

$$\text{Trajectory }4:\;\left[\begin{array}{cc} 5.3 & 4\\ 4 & 4\\ 4 & 5.3 \end{array}\right]$$

By solving the system of differential equations (Equations 7, 8), we obtained two EE values for a and b based on trajectory 1 and other initial conditions. We repeated this process for all four trajectories, resulting in four sets of EE values for a and b, respectively. These sets were then averaged at each time point, and the mean (μ^*^) and standard deviation (σ) were calculated across all time points according to Equations 5, 6. Finally, μ^*^ and σ of a were found to be 1910 and 3830, respectively, while those of b were 156 and 230, respectively. These findings suggest that variations in parameter ‘a’ exhibit a higher sensitivity towards the output ‘y’, and also unveil a more pronounced interaction and/or potentially nonlinear relationship between ‘a’ and the system variables. For detailed computational procedures of this application example, please refer to the MATLAB (MathWorks Inc., USA) programs provided alongside this article, named “myMorrisExample.m” and “myMorrisExampleODE.m”.

## 3 Results and discussion

In this study, the Morris SA was performed on 18 forward rate constants (all detailed in **Table 1**) and 14 backward rate constants (all detailed in **Table 2**) within Lazár’s model under low and intense illumination conditions. The SA procedure can be clarified through three main steps as follows:

**Table 1:** Description of forward rate constants used in Lazár’s model (2009) and their corresponding values used in simulations presented in **Figure 1**.

Rate Constant	Description & Electron Transport Reaction	Value (s^-1^)
k1f	Rate constant for light-induced charge separation between P680 and Q_A_, leading to the formation of P680^+^ and ${\text{Q}}_{\text{A}}^{-}$ P680(Pheo)Q_A_→P680^+^(Pheo) ${\text{Q}}_{\text{A}}^{-}$	2000
k2	Rate constant for the electron donation from the S0 state of the OEC to P680^+^ through ${\text{Y}}_{\text{Z}}^{+}$ during the S0-to-S1 transition S0P680^+^→S1P680	20000
k3	Rate constant for the electron donation from the S1 state of the OEC to P680^+^ through ${\text{Y}}_{\text{Z}}^{+}$ during the S1-to-S2 transition S1P680^+^→S2P680	10000
k4	Rate constant for the electron donation from the S2 state of the OEC to P680^+^ through ${\text{Y}}_{\text{Z}}^{+}$ during the S2-to-S3 transition S2P680^+^→S3P680	3330
k5	Rate constant for the electron donation from the S3 state of the OEC to P680^+^ through ${\text{Y}}_{\text{Z}}^{+}$ during the S3-to-S0 transition S3P680^+^→S0P680	1000
k6f	Rate constant governing the electron transfer from ${\text{Q}}_{\text{A}}^{-}$ to Q_B_ ${\text{Q}}_{\text{A}}^{-}$ Q_B_→Q_A_ ${\text{Q}}_{\text{B}}^{-}$	3500
k7f	Rate constant governing the electron transfer from ${\text{Q}}_{\text{A}}^{-}$ to ${\text{Q}}_{\text{B}}^{-}$ ${\text{Q}}_{\text{A}}^{-}$ ${\text{Q}}_{\text{B}}^{-}$ →Q_A_ ${\text{Q}}_{\text{B}}^{2-}$	1750
k8f	Rate constant for exchange involving the doubly reduced Q_B_ ( ${\text{Q}}_{\text{B}}^{2-}$ ) with an oxidized PQ molecule from the PQ pool Q_A_ ${\text{Q}}_{\text{B}}^{2-}$ +PQ+2H^+^→Q_A_Q_B_+PQH_2_	250
k9f	Rate constant for the oxidation of reduced PQ on the luminal side of cyt b_6_/f, leading to the transfer of one electron to haem b_L_ and one to haem *f* PQH_2_+fb_L_→PQ+f^−^ ${\text{b}}_{\text{L}}^{-}$ +2H^+^	100
k10f	Rate constant governing the electron transfer from ${\text{b}}_{\text{L}}^{-}$ to either oxidized or singly reduced haem b_H_ ${\text{b}}_{\text{L}}^{-}$ b_H_→b_L_ ${\text{b}}_{\text{H}}^{-}$ or ${\text{b}}_{\text{L}}^{-}$ ${\text{b}}_{\text{H}}^{-}$ →b_L_ ${\text{b}}_{\text{H}}^{2-}$	2300
k11f	Rate constant for the reduction of oxidized PQ on the stromal side of cyt b_6_/f by ${\text{b}}_{\text{H}}^{2-}$ ${\text{b}}_{\text{H}}^{2-}$ +PQ+2H^+^→b_H_+PQH_2_	100
k12f	Rate constant for the oxidation of reduced haem *f* by Pc^+^ f^−^Pc^+^→fPc	100
k13f	Rate constant for the reduction of either oxidized or singly reduced b_H_ by Fd^−^ b_H_+Fd^−^→ ${\text{b}}_{\text{H}}^{-}$ +Fd or ${\text{b}}_{\text{H}}^{-}$ +Fd^−^→ ${\text{b}}_{\text{H}}^{2-}$ +Fd	100
k14f	Rate constant for the reduction of either oxidized or singly reduced PQ by Fd^–^ PQ+Fd^−^→PQ^−^+Fd or PQ^−^+Fd^−^+2H^+^→PQH_2_+Fd	1
k15f	Rate constant for light-induced charge separation between P700 and F_B_, leading to the formation of P700^+^ and ${\text{F}}_{\text{B}}^{-}$ P700F_B_→P700+ ${\text{F}}_{\text{B}}^{-}$	2000
k16f	Rate constant governing the electron transfer from Pc to P700^+^ Pc+P700^+^→Pc^+^+P700	200
k17f	Rate constant governing the electron transfer from ${\text{F}}_{\text{B}}^{-}$ to Fd ${\text{F}}_{\text{B}}^{-}$ +Fd→F_B_+Fd^−^	200
k18f	Rate constant governing the electron transfer from ${\text{F}}_{\text{d}}^{-}$ to either actively oxidized or singly reduced FNR ${\text{F}}_{\text{d}}^{-}$ +FNR→Fd+FNR^−^ or ${\text{F}}_{\text{d}}^{-}$ +FNR^−^→Fd+FNR^2−^	5

**Table 2:** Description of backward rate constants used in Lazár’s model (2009) and their corresponding values used in simulations presented in **Figure 1**.

Rate Constant^*^	Electron Transport Reaction	Value (s^-1^)
k1b	P680^+^(Pheo) ${\text{Q}}_{\text{A}}^{-}$ →P680(Pheo)Q_A_	5000
k6b	Q_A_ ${\text{Q}}_{\text{B}}^{-}$ → ${\text{Q}}_{\text{A}}^{-}$ Q_B_	175
k7b	Q_A_ ${\text{Q}}_{\text{B}}^{2-}$ → ${\text{Q}}_{\text{A}}^{-}$ ${\text{Q}}_{\text{B}}^{-}$	35
k8b	Q_A_Q_B_+PQH_2_→Q_A_ ${\text{Q}}_{\text{B}}^{2-}$ +PQ+2H^+^	250
k9b	PQ+f^−^ ${\text{b}}_{\text{L}}^{-}$ +2H^+^→PQH_2_+fb_L_	10
k10b	b_L_ ${\text{b}}_{\text{H}}^{-}$ → ${\text{b}}_{\text{L}}^{-}$ b_H_ or b_L_ ${\text{b}}_{\text{H}}^{2-}$ → ${\text{b}}_{\text{L}}^{-}$ ${\text{b}}_{\text{H}}^{-}$	7
k11b	b_H_+PQH_2_→ ${\text{b}}_{\text{H}}^{2-}$ +PQ+2H^+^	10
k12b	fPc→f^−^Pc^+^	10
k13b	${\text{b}}_{\text{H}}^{-}$ +Fd→b_H_+Fd^−^ or ${\text{b}}_{\text{H}}^{2-}$ +Fd→ ${\text{b}}_{\text{H}}^{-}$ +Fd^−^	100
k14b	PQ^−^+Fd→PQ+Fd^−^ or PQH_2_+Fd→PQ^−^+Fd^−^+2H^+^	1
k15b	P700+ ${\text{F}}_{\text{B}}^{-}$ →P700F_B_	10000
k16b	Pc^+^+P700→Pc+P700^+^	10
k17b	F_B_+Fd^−^→ ${\text{F}}_{\text{B}}^{-}$ +Fd	10
k18b	Fd+FNR^−^→ ${\text{F}}_{\text{d}}^{-}$ +FNR or Fd+FNR^2−^→ ${\text{F}}_{\text{d}}^{-}$ +FNR^−^	5

^*^Rate constants ending with the letter ‘b’ represent the reverse processes of those characterized by rate constants ending with the letter ‘f’.

1. Optimal values for the model rate constants under each illumination condition can be determined by comparing the simulation results with the experimental curves.

2. Construction of X*. In this study, p is set to be 4. Therefore, Δ equals to 2/3. Consequently, X* is formed by randomly selecting values from the range (0, 1/3, 2/3, 1). Further, four trajectories (i.e., m = 4) are generated by randomly choosing four distinct values from this space. In this FI model, the calculation of Morris measures involves determining the mean (μ*) and standard deviation (σ) across all time points. The use of four trajectories in our study can meet the requirements for analyzing the FI model. However, it is worth noting that improved results are not necessarily obtained, as suggested by previous studies (Saltelli et al., 2006; Campolongo et al., 2007, 2011), when more trajectories are employed in simulations.

Construction of B*, calculation of EE, and calculation of the Morris measures. Following Equation 3, B* can be obtained based on X*. At this stage, values in the B* space do not represent the true values of rate constants. Mapping B* values to the real space of each rate constant involves scaling rate constants by 10%, 20%, and 30% (30% is only used in the SA for forward rate constants under the low light condition). For instance, scaling a rate constant by 10% creates an interval from 90% of the original value to 110% of the original value. After mapping B* to the real value interval of rate constants, in each trajectory, EE for each rate constant can be calculated. Subsequently, the mean (μ*) and standard deviation (σ) of these Morris measures can also be computed. The MATLAB code related to the Morris method of this study has been pushed to the open-source program platform at www.gitee.com. Readers can access and/or download these programs from the following website link: https://gitee.com/hui-lyu/sa-dl-code.

**Figure 1:**
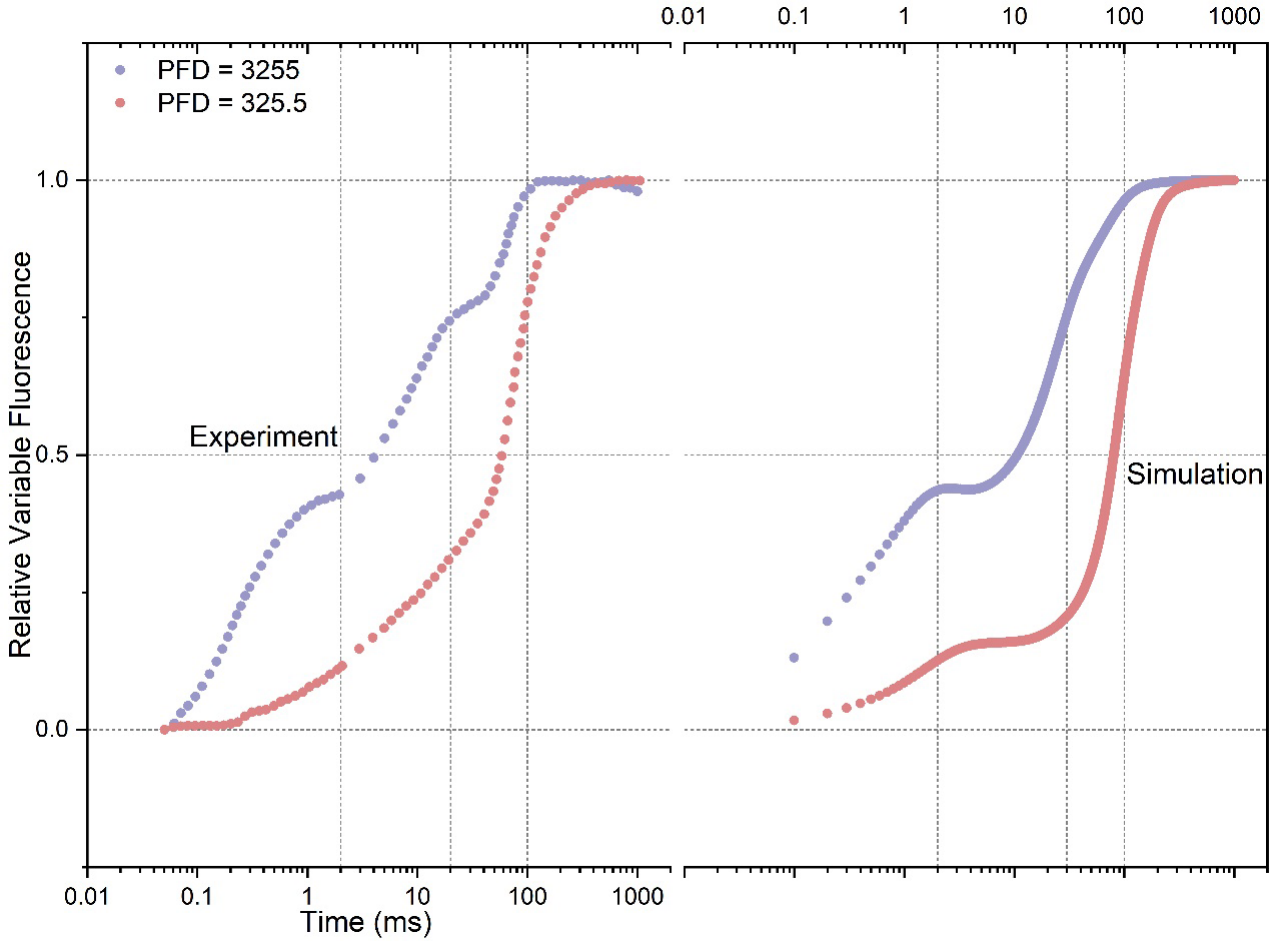
Comparison of experimental data with simulated rapid phases of Fluorescence Induction (FI) curves under both intense (PFD: 3255 photons m^-2^ s^-1^) and low (PFD: 325.5 photons m^-2^ s^-1^) illumination conditions. PFD represents Photon Flux Density. The experimental data are from Strasser et al. (1995).

Upon the conditions of intense illumination (PFD: 3255 photons m^-2^ s-^1^) and low illumination (PFD: 325.5 photons m^-2^ s^-1^), **Figure 1** illustrates a comparison between model outputs and experimental curves. In the condition of high illumination, the experimental curve shows distinct O-J-I-P transitions, where the J point occurs at around 2 ms, I at around 20 ms, and P at around 200 ms. Notably, the simulated curve agrees well with the experimental curve, with the J (~ 2 ms), I (~ 20 ms), and P (~ 200 ms) points occurring at similar time intervals. However, the simulated curve exhibits a more pronounced plateau at the J inflection point compared to the experimental curve, whilst the I inflection point is much less evident. Additionally, the amplitude at the O point in the simulated curve exceeds that of the experimental curve, indicating a higher simulated fluorescence intensity immediately upon light activation. In the condition of low illumination, the J inflection point in the experimental curve occurs much faster (~ 0.3 ms) but with a significantly reduced amplitude compared to the curve measured under intense illumination. Simultaneously, the I inflection point disappears in the experimental curve, and the P point emerges at approximately 200 ms. In the simulated curve, there is also a noticeable reduction in the amplitude of the plateau at the J inflection point, which agrees well with the pattern observed in the experimental curve. However, it is worth noting that the plateau occurs at a delayed time point (~ 2 ms) in the simulated curve. Similar to the observed pattern in the measured curve, the simulated curve also illustrates the disappearance of the I inflection point, and the occurrence of the P point is also delayed. In general, the simulated curves can qualitatively reproduce the characteristic features observed in the experimental curves.

It’s worth noting that achieving a precise reproduction of variables of interest measured during photosynthesis remains a challenge for any photosynthetic model. This challenge may arise from the complexity of the models and the limited number of experimentally validated determinations for rate constants, which makes the models highly underdetermined. Moreover, despite their complexity, most models likely do not include enough detail about real physiological processes that could dynamically modify the parameter values. Another possibility is that our understanding of the fundamental properties of chl *a* fluorescence remains insufficient, thereby impeding accurate simulation of experimental data by models. These suggest that the simulated results may only qualitatively reproduce the characteristics of experimental FI curves, even in comprehensively constructed photosynthesis models that encompass nearly the entire leave-based photosynthetic reactions, as shown in studies by Laisk et al. (2006) (Laisk et al., 2006) and Zhu et al. (2013) (Zhu et al., 2013).

**Figure 2:**
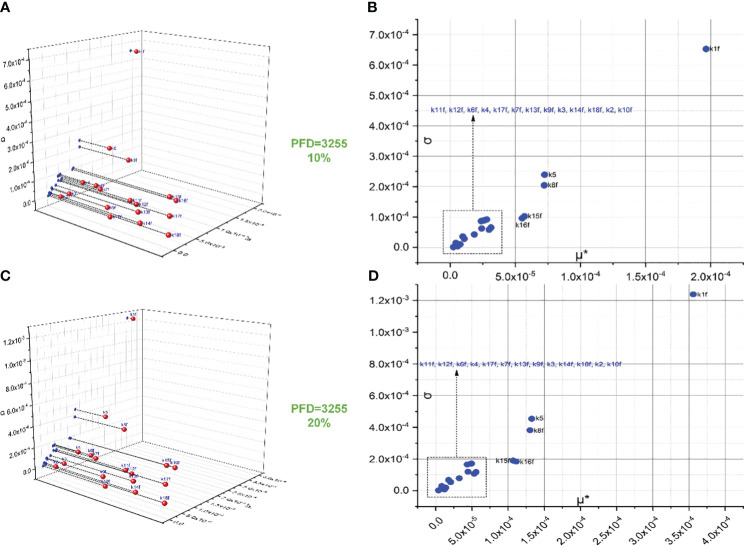
Screening of 18 forward rate constants scaled by 10% **(B)** and 20% **(D)** of their original values under intense illumination. **(A, C)** represent the 3D versions of the corresponding 2D presentations.

**Figure 3:**
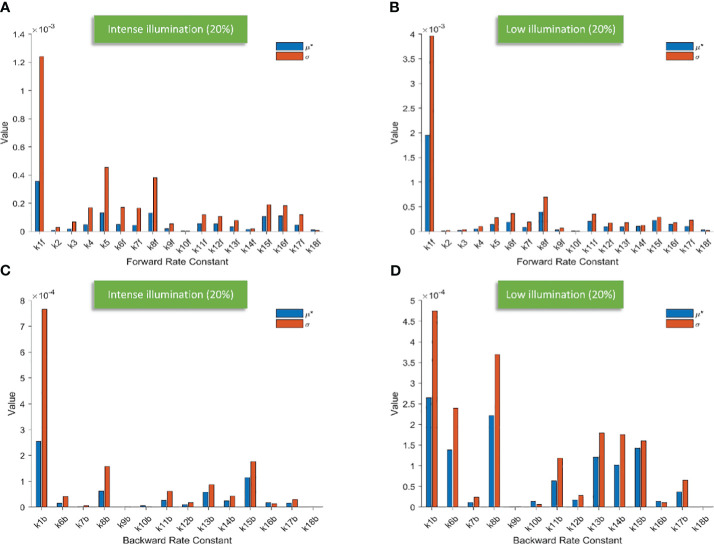
Bar charts illustrating the screening results of 18 forward rate constants scaled by 20% of their original values under intense illumination **(A)** and under low illumination **(B)**; bar charts illustrating the screening results of 14 backward rate constants scaled by 20% of their original values under intense illumination **(C)** and under low illumination **(D)**.

The screening of 18 forward rate constants under intense illumination is presented in **Figure 2**. Rate constants scaled by 10% of their original values are shown in **Figures 2A, B**, and those scaled by 20% of their original values are shown in **Figures 2C, D**. **Figures 2A, C** are the 3D versions of the corresponding 2D presentations. The sorting results for those scaled by 20% are also depicted in bar charts as shown in **Figure 3A**. Different scales are used to the SA to reduce the pronounced impact of certain rate constants, especially when the scale is very small. This is because the effects of rate constants on shaping the FI curve would be saturated when the scale is increased. Therefore, this treatment can help reveal the genuine importance of rate constants. By employing the Morris SA, for both 10% and 20% scales, we can sort all the forward rate constants based on their importance, as derived from the values of μ*. The rate constants are sequenced in descending order as follows: (1) k1f, describing the reaction rate of P680(Pheo)Q_A_ → P680^+^(Pheo) ${\text{Q}}_{\text{A}}^{-}$ ; (2) k5, describing the rate constant for electron donation from the S3 state of the OEC to P680^+^ during the S3−to−S0 transition; (3) k8f, describing the rate constant for exchange involving the doubly reduced Q_B_ with an oxidized PQ molecule from the PQ pool; (4) k15f (10%), describing the rate constant for electron transfer from Plastocyanin (Pc) to P700^+^ or k16f (20%), describing the rate constant for light-induced charge separation between P700 and F_B_, thereby leading to the formation of P700^+^ and reduced F_B_; (5) k16f (10%) or k15f (20%); (6) k11f, describing the rate constant for the reduction of PQ on the stromal side of Cytb_6_/f by doubly reduced haem b_H_; (7) k12f, describing the rate constant for the oxidation of reduced haem *f* by Pc^+^; (8) k6f, describing the rate constant for electron transfer from reduced Q_A_ to Q_B_; (9) k4, describing the rate constant for electron donation from the S2 state of the OEC to P680^+^ during the S2−to−S3 transition; (10) k17f, describing the rate constant for electron transfer from reduced F_B_ to Fd; (11) k7f, describing the rate constant for electron transfer from reduced Q_A_ to singly reduced Q_B_; (12) k13f, describing the rate constant for the reduction of either oxidized or singly reduced haem b_H_ by Fd^–^; (13) k9f, describing the rate constant for the oxidation of reduced PQ on the luminal side of Cytb_6_/f, thereby leading to the transfer of one electron to haem b_L_ and one to haem *f*; (14) k3, describing the rate constant for electron donation from the S1 state of the OEC to P680^+^ during the S1−to−S2 transition; (15) k14f, describing the rate constant for the reduction of either oxidized or singly reduced PQ by Fd^–^; (16) k18f, describing the rate constant for electron transfer from Fd^–^ to either actively oxidized or singly reduced FNR; (17) k2, describing the rate constant for electron donation from the S0 state of the OEC to P680^+^ during the S0−to−S1 transition; and (18) k10f, describing the rate constant for electron transfer from reduced haem b_L_ to either oxidized or singly reduced haem b_H_.

Conceivably, k1f, which represents the primary charge separation in PSII, is the most significant parameter in the entire photosynthetic system. Moreover, the importance of donor-side-based k5, representing rate constant for the electron donation from the S3 state of the OEC to P680^+^ through ${\text{Y}}_{\text{Z}}^{+}$ during the S3-to-S0 transition, is higher than that of acceptor-side-based k8f, which represents the rate constant for exchange involving the doubly reduced Q_B_ ( ${\text{Q}}_{\text{B}}^{2-}$ ) with an oxidized PQ molecule from the PQ pool. Following k8f, the importance of k15f, representing the rate constant for light-induced charge separation between P700 and F_B_, exceeds that of k16f, which represents the rate constant governing the electron transfer from PC to P700^+^. Interestingly, around PSI, the importance of donor-side-based k16f is also higher than that of acceptor-side-based k17f, which represents the rate constant governing the electron transfer from reduced F_B_ to F_d_. However, rate constants for cyclic electron reactions, such as k13f (the rate constant for the reduction of either oxidized or singly reduced b_H_ by Fd^–^) and k14f (the rate constant for the reduction of either oxidized or singly reduced PQ by Fd^–^) are shown to be less significant than empirically suggested by some modelling studies (Li et al., 2021). By employing the Morris SA, we can also sort all the forward rate constants based on their linearity and/or potential reactions with other parameters, as derived from the values of σ. Notably, k1f occupies the highest rank, followed by k5, k8f, k15f, and k16f. This ranking corresponds to the importance sequence for these rate constants, indicating that a rate constant with higher significance is more likely to exhibit nonlinearity and/or interact with other parameters.

**Figure 4:**
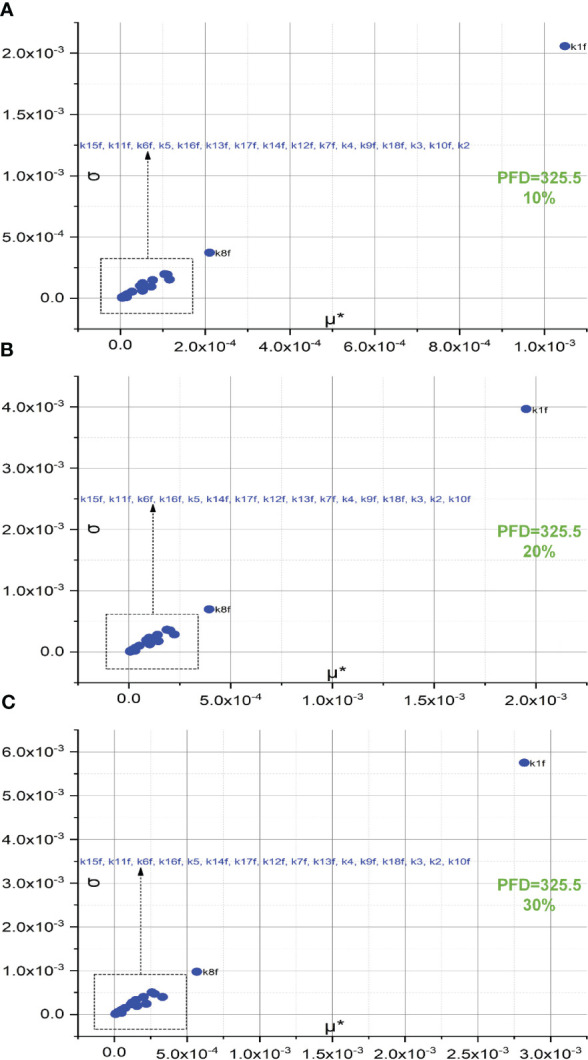
Screening of 18 forward rate constants scaled by 10% **(A)**, 20% **(B)**, and 30% **(C)** of their original values under low illumination.

**Figure 4** represents the screening of 18 forward rate constants under low illumination. Rate constants scaled by 10%, 20%, and 30% of their original values are respectively shown in **Figures 4A–C**. The sorting results for those scaled by 20% are also illustrated in bar charts as shown in **Figure 3B**. To simulate FI curve under low illumination, we adjust the values for k1f, k1b, k15f, and k15b to one-tenth of their original values under intense illumination, while keeping all other values of rate constants unchanged. Surprisingly, the ranking sequence for 18 forward rate constants noticeably varies when those specific rate constant values are altered. In specific, the leading group in the ranking sequence comprises only k1f and k8f. The composition of the less significant group varies from 10% to 20% scales, while the composition remains identical for the 30% scale as compared to the 20% scale. This suggests that the effects of these rate constants on shaping FI curve reach saturation at the 20% level. The forward rate constants in the less significant group for both 20% and 30% levels include k15f, k11f, k6f, k16f, k5, k14f, k7f, k12f, k13f, k7f, k4, k9f, k18f, k3, k2, and k10f. These constants are sorted based on the importance sequence derived from the values of μ* (see **Figures 4B, C**). It is worth noting that k1f and k8f are the most influential rate constants when simulating FI curves under both intense and low illumination. For the precise classification of forward rate constants in the less significant group for 10%, refer to **Figure 4A**.

**Figure 5:**
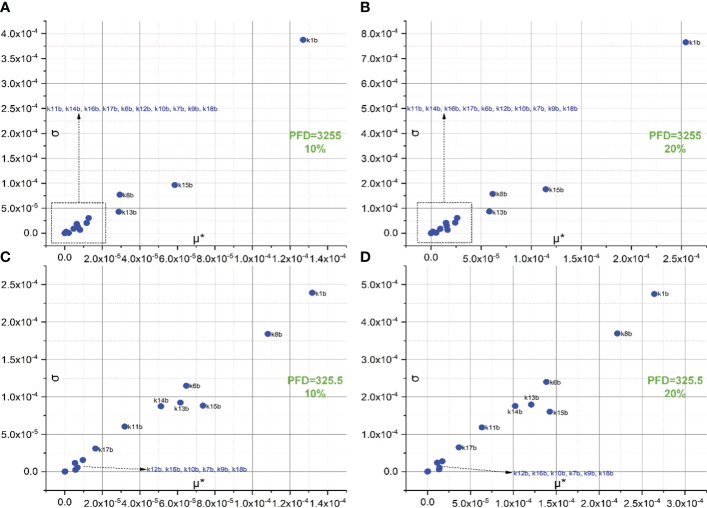
Screening of 14 backward rate constants scaled by 10% of their original values under both intense **(A)** and low **(C)** illumination conditions and 20% under both intense **(B)** and low **(D)** illumination conditions.

**Figure 5** illustrates the screening of 14 backward rate constants scaled by 10% and 20% in panels A and B under intense illumination, and panels C (10% scale) and D (20% scale) under low illumination. The sorting results for those scaled by 20% are also portrayed in bar charts as shown in **Figure 3C** under intense illumination and **Figure 3D** under low illumination. Overall, the impact on shaping the FI curve due to the forward rate constants is more significant than that of backward rate constants as expected. This is evident in the magnitude of μ* for forward rate constants compared to backward rate constants under corresponding illumination conditions. For intense illumination, the ranking sequence remains identical for both the 10% and 20% scales. The leading group consists of k1b, k15b, k8b, and k13b, followed by a less significant group including k11b, k14b, k16b, k17b, k6b, k12b, k10b, k7b, k9b, and k18b. Similarly, for low illumination, the ranking sequence also remains unchanged for both the 10% and 20% scales. The leading group consists of k1b, k8b, k15b, k6b, k13b, k14b, k11b, and k17b, followed by a less significant group including k12b, k16b, k10b, k7b, k9b, k18b. However, it is also observable that the ranking sequence varies significantly under intense illumination compared to low illumination. Moreover, it is worth noting that k1b, k8b, k13b, and k15b are the most influential rate constants when simulating FI curves under both intense and low illumination. All ranking sequences for both forward rate constants and backward rate constants under varying light conditions are provided in **Table 3**.

**Table 3:** Orderings of 18 forward rate constants and 14 backward rate constants based on their importance, as derived from values of μ*.

Order	Intense light (forward rate constant)	Low light (forward rate constant)	Intense light (backward rate constant)	Low light (backward rate constant)
1	k1f	k1f	k1b	k1b
2	k5	k8f	k15b	k8b
3	k8f	k15f	k8b	k15b
4	k16f	k11f	k13b	k6b
5	k15f	k6f	k11b	k13b
6	k11f	k16f	k14b	k14b
7	k12f	k5	k16b	k11b
8	k6f	k14f	k17b	k17b
9	k4	k17f	k6b	k12b
10	k17f	k12f	k12b	k16b
11	k7f	k7f	k10b	k10b
12	k13f	k13f	k7b	k7b
13	k9f	k4	k9b	k9b
14	k3	k9f	k18b	k18b
15	k14f	k18f		
16	k18f	k3		
17	k2	k2		
18	k10f	k10f		

**Figure 6:**
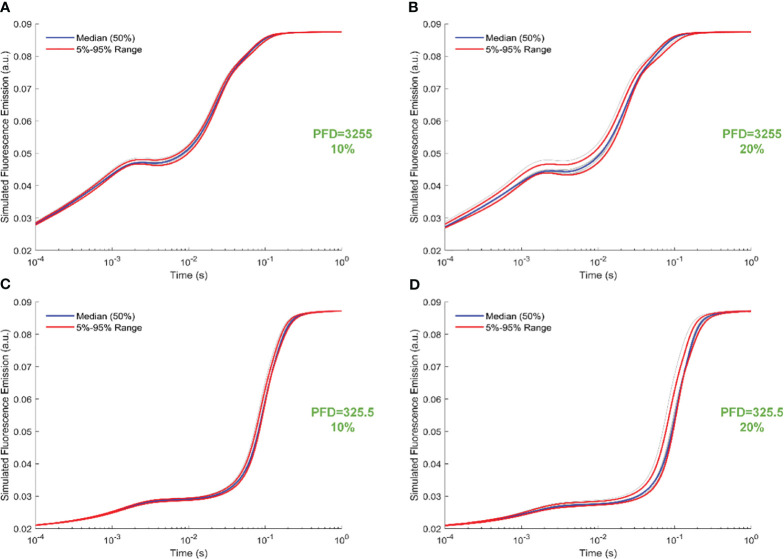
Trajectory-sampling-based FI simulations with forward rate constants scaled by 10% and 20% in **(A, B)** under intense illumination, and **(C)** (10% scale) and **(D)** (20% scale) under low illumination. The fourth trajectory is utilized to generate these figures.

**Figure 7:**
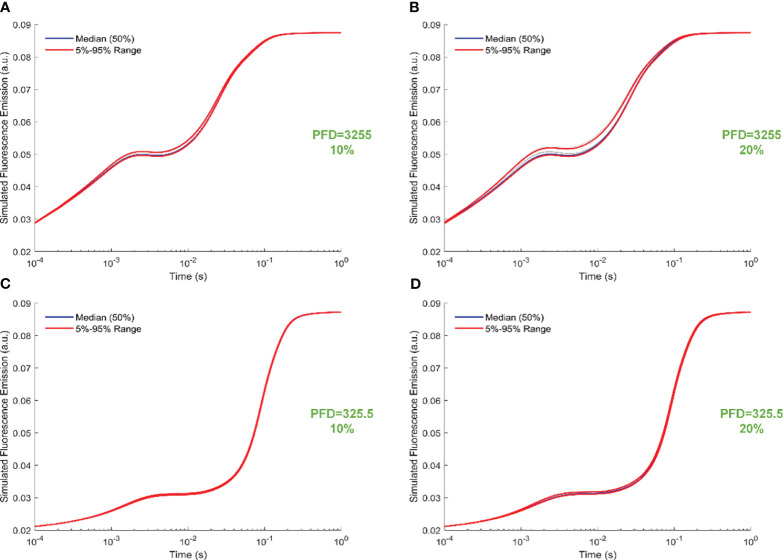
Trajectory-sampling-based FI simulations with backward rate constants scaled by 10% and 20% in **(A, B)** under intense illumination, and **(C)** (10% scale) and **(D)** (20% scale) under low illumination. The fourth trajectory is utilized to generate these figures.

**Figure 6** illustrates trajectory-sampling-based FI simulations for forward rate constants scaled by 10% and 20% in panels A and B under intense illumination, and panels C (10% scale) and D (20% scale) under low illumination. Additionally, the 5th, 50th, and 95th percentiles are specifically highlighted in the simulated 19 curves across all panels. Overall, the impact of forward rate constants, when uniformly scaled, is more noticeable in shaping the simulated FI curves under intense illumination than low illumination. At the same time, the effect of forward rate constants, when uniformly illuminated, is more noticeable in shaping the simulated FI curves under the 20% scale compared to the 10% scale. Specifically, for the 20% scale under intense illumination, the simulated curves undergo changes throughout the entire course of O-J-I-P transients. In contrast, for the 20% scale under low illumination, the alterations in the simulated curves occur mainly during the J-I-P phase. Furthermore, **Figure 7** illustrates trajectory-sampling-based FI simulations for backward rate constants scaled by 10% and 20% in panels A and B under intense illumination, and panels C (10% scale) and D (20% scale) under low illumination. In any case, the alterations in the simulated curves are much less pronounced than those observed for forward rate constants. Specifically, for low illumination at10% and 20% scales, the alterations in the simulated curves are nearly unnoticeable. Under intense illumination at 10% and 20% scales, the changes in the simulated curves primarily occur during the J-I phase.

**Figure 8:**
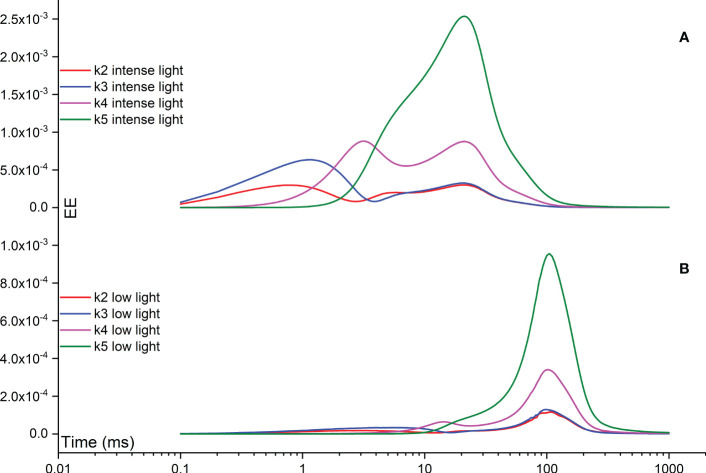
Time courses illustrating the Elementary Effect (EE) arising from rate constants related to the functionality of OEC (k2, k3, k4, and k5) under both intense **(A)** and low **(B)** illumination conditions. The figures are generated based on the fourth trajectory.

**Figure 9:**
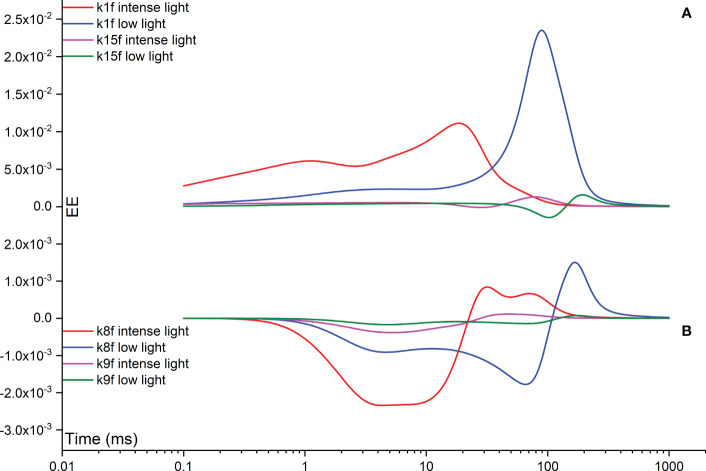
Time courses illustrating the Elementary Effect (EE) arising from forward rate constants k1f **(A)**, k15f **(A)**, k8f **(B)**, and k9f **(B)** under both intense and low illumination conditions. The figures are generated based on the fourth trajectory.

The Morris measure, EE, directly focuses on the net variation of simulated FI curves resulting from a random change in a specific rate constant. **Figure 8** illustrates temporal variations induced by rate constants related to the functionality of OEC, specifically regulated by rate constants k2, k3, k4, and k5. Notably, under intense illumination, the most significant deviation is attributed to k5, followed by k4, k3, and k2 in sequence. The maximum deviation induced by k5 occurs at approximately 20 ms, coinciding with the appearance of the I point. For k4​, two maximum deviations occur: one at the J point and another at the I point. Conversely, k3 peaks at about 1 ms, corresponding to the appearance of the J point, while k2​ peaks at a slightly earlier time point. Under low illumination, nearly all maximum deviations shift to a later time point at approximately 100 ms, aligning with the appearance of the P point. In other words, when illumination is reduced, the turnover of the OEC can indirectly affect the P point of the simulated FI dynamics. This occurs because PSII-donor-side limitation can decelerate the electron transport rate, leading to a delayed occurrence of the P point. In some cases, the P point may not occur at all, particularly when the FNR complex is activated within the model. However, the deviation in k5 still maintains its status as the highest, followed sequentially by k4, k3, and k2, similar to the pattern observed under intense illumination. The significance in shaping the FI curve for k1f is much higher than that of k15f under both intense and low illumination conditions. This perspective is also supported by **Figure 9**, which agrees well with the view that PSII is the major contributor for generating the FI curve. Surprisingly, under both intense and low illumination conditions, shaping the FI curve for k8f is more significant than for k9f which represents the rate limiting step in linear electron transport. The growing significance of k8f compared to k9f suggests that k8f, representing the rate constant for the exchange involving the doubly reduced Q_B_ with an oxidized PQ molecule, might be the key factor, at least in our model analysis, in regulating the electron transport from PSII to Cytb_6_/f. In **Figure 9B**, both negative and positive peaks are noticeable in the temporal EE curve for k8f under both intense and low illumination conditions. Additionally, in **Figure 9**, both negative and positive maximum deviations shift to occur at an earlier time point under low illumination compared to intense illumination.

**Figure 10:**
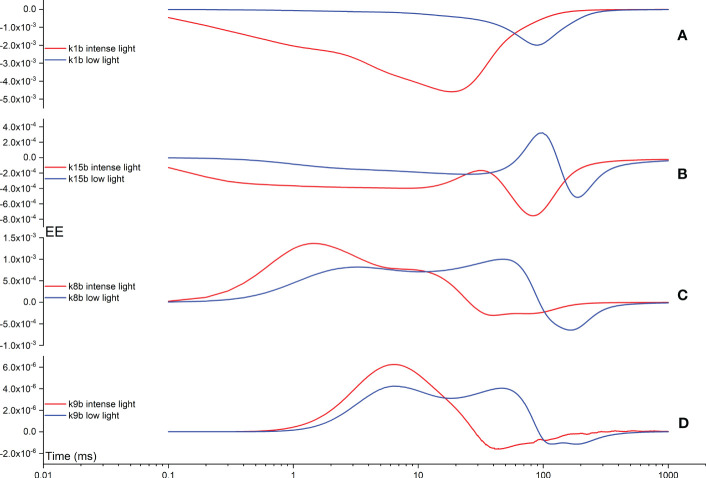
Time courses illustrating the Elementary Effect (EE) arising from backward rate constants k1b **(A)**, k15b **(B)**, k8b **(C)**, and k9b **(D)** under both intense and low illumination conditions. The figures are generated based on the fourth trajectory.

In the following analysis, we further explore the impact of backward rate constants on shaping the FI curves. In **Figure 10A**, the k1b EE curve displays a maximum deviation at approximately 20 ms, corresponding to the appearance of the I point during intense illumination. Conversely, under low illumination, a maximum deviation is observed at approximately 100 ms in the k1b EE curve, albeit with a lower amplitude compared to that observed under intense illumination. In **Figure 10B**, the k15b EE curve exhibits a two-peak pattern in opposite directions during intense illumination. This pattern remains consistent during low illumination, albeit with all maximum deviations shifted to occur at an earlier time point. Generally, the influence of k15b on shaping the FI curves is notable during the time interval between the I point and P point, or even beyond the P point. In **Figure 10C**, the k8b EE curve exhibits a two-peak pattern in the same direction during intense illumination. This pattern persists during low illumination as well, although all maximum deviations are shifted to occur at an earlier time point. The influence of k8b on shaping the FI curves is generally observed during the time interval between the J point and P point, or even beyond the P point. Ultimately, in **Figure 10D**, the k9b EE curve exhibits a two-peak pattern in opposite directions during intense illumination. Conversely, under low illumination, it demonstrates a two-peak pattern in the same direction, yet with all maximum deviations shifted to occur at an earlier time point. Generally, the influence of k9b on shaping the FI curves is observed during the time interval between the J point and P point.

Previously, Lazár et al. (2005) employed a method of Metabolic Control Analysis (MCA) to evaluate the importance of photosynthetic reactions in controlling the level of P point within a PSII model that explores the activity of Cytochrome b_559_. Additionally, Ebenhöh et al. (2011) employed a local SA to evaluate the importance of four parameters in controlling the steady-state systematic behavior, with a specific focus on the nonphotochemical quenching of chl *a* fluorescence. Moreover, Zhu et al. (2013) also employed a local SA to evaluate the significance of parameters in controlling the steady-state systematic behavior of the Calvin-Benson-Basham (CBB) cycle. By using the local SA method in the mentioned studies, the authors can only manually adjust the parameters of interest around a specific value point. On the contrary, a global SA tool such as the Morris method can randomly alter the values of given parameters across the entire input space. Nonetheless, it is important to note that the Morris method has its own limitations. For instance, this global SA technique can highlight which factor has a high tendency to exhibit nonlinearity and/or interact with other parameters, but it cannot specifically identify which factor truly correlates with the given factor. Additionally, the results of SA employing the Morris method may depend on the structural feature of the model under investigation. In this research, Lazár’s model is used for our case study. If another model with a different structure is used, the SA results may vary accordingly. However, by utilizing the Morris SA, researchers can systematically screen the shared key factors influencing outcomes from different models, provided that all investigated models are available. In principle, after deriving SA conclusions for model parameters via the Morris method, we can enhance the model itself by concentrating on highly sensitive parameters. This could involve fine-tuning these parameters to better match observed behaviors or trends in the output data. Furthermore, we can employ the SA findings to prioritize additional investigations or experiments aimed at validating the model’s behavior across various parameter settings. Ultimately, utilizing the insights obtained from the Morris SA enables targeted refinement of the model, thereby improving its accuracy and predictive capabilities. To the best of our knowledge, the integration of a global SA approach with a FI model has not been explored in previous publications. In conclusion, the Morris SA algorithm demonstrated its capability to operate under varying conditions and produce reliable results. By utilizing these findings, we can further enhance the accuracy and reliability of photosynthesis models, thereby advancing our understanding of the complex processes involved in photosynthetic systems.

## 4 Concluding remarks

In this study, we utilized the Morris method, an efficient and widely used SA tool, to evaluate the significance of rate constants on shaping FI curves under varying illumination regimes. This global SA was performed on an existing FI model, providing an in-depth understanding of the role of reactions, particularly those related to the electron transport chain, in shaping the fluorescence dynamics and elucidating their impact. To determine optimal values for the model rate constants under different illumination conditions, we compared simulated results with experimental data. Subsequently, we conducted the Morris SA to further analyze these rate constants. By using the Morris method to analyze Lazár’s model, we observed significant variations in the ordering of all rate constants when specific rate constant values were altered. Ultimately, we clarified the net variations in simulated FI curves resulting from random changes in these specific rate constants. In summary, our findings, derived from a global SA tool, provide a novel outlook on screening the photosynthetic reactions that can notably impact the rapid FI curves under various illumination conditions.

## Data availability statement

The original contributions presented in the study are included in the article/**Supplementary Material**. Further inquiries can be directed to the corresponding authors.

## Author contributions

HL: Conceptualization, Formal analysis, Methodology, Writing – original draft, Writing – review & editing. YL: Data curation, Funding acquisition, Visualization, Writing – original draft, Writing – review & editing. GL: Writing – original draft, Writing – review & editing.
